# Ice nucleation active bacteria metabolites as antibiofilm agent to control *Aeromonas hydrophila* and *Streptococcus agalactiae* infections in Aquaculture

**DOI:** 10.1186/s13104-024-06821-9

**Published:** 2024-06-17

**Authors:** Jessica Kurniawan, Diana Elizabeth Waturangi, Pande Gde Sasmita Julyantoro, Nurmaya Papuangan

**Affiliations:** 1 Department of Biotechnology, Faculty of Biotechnology, Atma Jaya Catholic University of Indonesia, Jalan Jenderal Sudirman 51, Jakarta, 12930 Indonesia; 2 Department of Aquatic Resources Management, Faculty of Marine Science and Fisheries, University of Udayana, Denpasar, Bali, 80361 Indonesia; 3 Department of Biology Education, Faculty of Teacher Training and Education, Khairun University, Ternate, 97728 Indonesia

**Keywords:** Biofilms, Fish pathogenic bacteria, Ice nucleation active bacteria, Antibiofilm

## Abstract

**Objectives:**

The aim of this study was to quantify and identify metabolites of Ice Nucleation Active (INA) bacteria as an anti-biofilm agent against biofilms of fish pathogens such as *Aeromonas hydrophila* and *Streptococcus agalactiae.*

**Results:**

Ice nucleation active bacteria, which have the ability to catalyze ice nucleation, isolated from rainwater in previous studies, were used. All INA isolates were tested in several assays, including the antimicrobial test, which uses streptomycin as the positive control and none of the isolates were found positive in the antimicrobial test. As for the quorum quenching assay, it was found that four out of ten isolates were able to disturb the communication system in *Chromobacterium violaceum* wild type, which was used as the indicator bacteria. On the next assay, all ten isolates were tested for Biofilm Inhibition and Destruction and showed anti-biofilm activity with the highest percentage inhibition of 33.49% by isolate A40 against *A. hydrophila* and 77.26% by isolate A19 against *S. agalactiae*. C1 performed the highest destruction against *A. hydrophila* and *S. agalactiae*, with percentages of 32.11% and 51.88%, respectively. As for the GC-MS analysis, supernatants of INA bacteria contain bioactive compounds such as sarcosine and fatty acids, which are known to have antibiofilm activity against several biofilm-forming bacteria. Through 16s rRNA sequencing, identified bacteria are from the *Pantoea, Enterobacter*, and *Acinetobacter* genera. As for the conclusion, ice nucleation active bacteria metabolites tested showed positive results against pathogenic bacteria *Aeromonas hydrophila* and *Streptococcus agalactiae* in destructing and inhibiting biofilm growth.

**Supplementary Information:**

The online version contains supplementary material available at 10.1186/s13104-024-06821-9.

## Introduction

The high amount 71% of Earth’s surface is covered with water, and the fisheries sector is considered to have a massive opportunity for fish production. Based on the data collected by the Food and Agriculture Organization, in 2020, the world has gained 177.8 million tons of fish coming through both fisheries and aquaculture, contributing to one-third of the global food production [1–3]. However, due to the intensive farming system, problems such as disease infection in fishes by pathogenic bacteria, *Aeromonas hydrophila* and *Streptococcus agalactiae*, may arise and cause serious repercussions such as failure of fish production that further lead to economic loss. These pathogens are able to form a matrix biofilm, which will attach irreversibly to an inanimate or solid surface to protect themselves against unfavorable environmental conditions [4–6].

To eradicate bacterial infections in aquaculture, antibiotics that are administered through their foods, baths, or by injection are widely used to control disease infections, but prolonged exposure of antibiotics to these pathogens may cause resistance as the biofilms formed block the access to the bacterial communities; therefore, it may lead to multidrug resistance. As resistance is triggered, it may easily spread among other aquatic microbial communities through horizontal gene transfer, posing greater health risks to human health as some pathogens are zoonotic [7–9].

Several considered alternative strategies used due to the emergence of antimicrobial resistance in aquaculture are vaccination, the use of probiotics and prebiotics, chicken egg yolk antibodies, bacteriophages, and quorum quenching, which is included in the antibiofilm activity [8]. Natural metabolite compounds from Ice Nucleation Active (INA) bacteria could be used as an alternative solution in antibiofilm activity; INA is a group of bacteria that are able to catalyze ice nucleation in temperatures just below 0 °C, which makes these bacteria able to withstand very low temperatures [10]. Guerra et al. (2022) and Goel et al. (2022) reported INA bacteria, *Klebsiella pneumoniae* and *Pantoea agglomerans*, could be used as an antibiofilm agent against *Vibrio* spp., as they produced metabolite compounds known to be monosaccharides and alpha-amylase respectively. Therefore, it is necessary to find natural metabolite compounds that are able to pose as an anti-biofilm agent [11, 12].

In this study, we identified and quantified the metabolites of INA bacteria, *Pantoea, Enterobacter*, and *Acinetobacter* genera by detecting its activity as an anti-biofilm agent against biofilms of fish pathogenic bacteria, *A. hydrophila* and *S. agalactiae*. As an anti-biofilm agent, these identified metabolites have shown several mechanisms, such as inhibition of bacterial surface adhesion and biofilm maturation, destruction of Biofilm, and quorum quenching [13].

## Main text

### Methods

#### Bacterial cultivation

Ten INA bacterial isolates (A19, A30, A32, A40, B10, B212, C1, J70, J73, and T125) from a previous study Stephanie and Waturangi (2011) [14] were cultured on Luria-Bertani Agar (LA) (Oxoid, Basingstoke, United Kingdom) and incubated at 30 °C for 24 h. *C. violaceum* wild type and *C. violaceum* 026 used as indicator bacteria were cultured on LA and incubated at 28 °C for 48 h. Fish pathogens obtained from the Department of Aquaculture, Faculty of Fisheries and Marine Science, IPB University, *A. hydrophila* and *S. agalactiae*, were also cultured on LA and incubated at 28 °C and 37 °C, respectively, for 24 h.

#### Production of bacterial metabolite

The ten isolates of INA bacteria (A19, A30, A32, A40, B10, B212, C1, J70, J73, and T125) were inoculated in 100 mL of Luria Broth (LB) and incubated at 30 °C, 125 rpm, for 48 h. *Pantoea agglomerans* suspension was then centrifuged at 5752 xg for 20 min. The cell-free metabolite obtained was then concentrated 5x using a freeze-dryer [15].

#### Detection of antimicrobial activity

The purpose of this assay is to confirm the antibiofilm activity and not because of antimicrobial activity, the negative result of antimicrobial activity are going to be continued with antibiofilm assay. This assay was conducted using the agar well diffusion method [16], and fish pathogens were inoculated into LB and incubated at optimum temperatures at 150 rpm for 24 h using an incubator shaker. Both fish pathogenic suspensions were diluted with sterile LB until the absorbance value reached 0.132 at 600 nm. 100 µL of pathogenic cultures were streaked on Mueller-Hinton Agar (MHA). Using a sterile cork borer, wells were made, and each well was filled with 100 µL of INA bacteria metabolites to each well [17]. Streptomycin (Merck; 10 mg/mL) was used as a positive control, while sterile LB was used as a negative control. These plates were then incubated at the optimum temperature of each pathogenic bacteria and this assay was done in triplicate [18].

#### Detection of anti-quorum sensing activity

In this assay, *C. violaceum* was used as indicator bacteria for assessment of anti quorum sensing activity, wild type of this strain was inoculated into LB, then incubated at 28 °C, 125 rpm, for 24 h. The optical density of bacterial suspension was adjusted to 0.132 at 600 nm, and then 100 µL of the suspension was streaked onto Brain Heart Infusion Agar (BHIA) (Oxoid, Basingstoke, United Kingdom). Wells was formed using a sterile cork borer and then filled with 100 µL of INA bacteria metabolites [19]. Streptomycin (10 mg/mL) was used as a positive control, and sterile LB was used as a negative control. The plates were then incubated at 28 °C for 24 h and this assay was done in triplicate [20].

#### Validation of quorum sensing inhibition

*C. violaceum* 026 was inoculated into sterile LB and incubated at 28 °C for 24 h at 125 rpm. The absorbance value was adjusted to 0.1 at 540 nm. The culture of *C. violaceum* 026 was then transferred to microtubes, and INA metabolites were added at a 1:1 ratio. 1 µmol/mL of Hexanoyl Homoserine Lactone (HHL; Oxoid) were added to the mixture and incubated at 28 °C for 24 h. Microtubes were then centrifuged at 1000 xg for 20 min, discarded supernatant, and 1 mL of DMSO was added. Microtube were centrifuged once more with the same condition to solubilize violacein and remove unwanted cells, and the supernatant was measured at 540 nm [19] rajivgandhi. The positive control used was a mixture of *C. violaceum* 026 and HHL without INA metabolite, while the negative control used was sterile LB. This assay was done in triplicates .

#### Biofilm inhibition and destruction assay

This assay was done using two mechanisms: inhibition and destruction. Fish pathogens were incubated into Brain Heart Infusion Broth (BHIB) (Oxoid, Basingstoke, United Kingdom) at each optimum temperature for 24 h. The optical density of each pathogen was adjusted to 0.132 at 600 nm. For inhibition assay, 100 µL of pathogenic bacteria suspension was transferred into 96 wells of polystyrene microplate followed by adding 100 µL of INA metabolite and then incubated at optimal temperatures of each pathogenic bacteria for 24 h. For the destruction assay, 100 µL of the pathogenic bacteria suspension was transferred into a polystyrene microplate and then incubated at each optimal temperature for 24 h. 100 µL of INA metabolite was added to each well, then continued overnight incubation at the same temperature [20]. For both assays, positive control was cultures of fish pathogens while negative control used was sterile BHIB.

After incubation, planktonic cells and media were discarded, each well was rinsed with water and air-dried for 30 min before adding 200 µL of 0.4% (b/v) crystal violet to each well and letting it stain for another 30 min. Crystal violet was discarded and stained cells were rinsed with water to remove remaining crystal violet before air-drying for 30 min [20] 200 µL of ethanol was added to each well, then measure absorbance at 595 nm using a microplate reader (TECAN M200 PRO) [21]. Both assays were done in triplicates and the percentage of both assays was calculated using the formula below [21]:

$$\begin{array}{l}Inhibition\,or\,Destruction\\=\frac{OD\,positive\,control- OD\,Sample}{OD\,positive\,control}\,\times\,100\%\end{array}$$

#### Microscopic observation of biofilm

Biofilms were observed under the microscope. Pathogenic bacteria were inoculated into a sterile BHIB and incubated at its optimum temperature for 24 h. The optical density of the bacterial culture was adjusted to 0.132 at 600 nm and let to grow on a cover glass for 24 h. After incubation, 100 µL of the selected metabolite was added to the cover glass and incubated for another 24 h [21].

For light microscope observation, the cover glass was rinsed with 1mL of distilled water and stained with 1 mL of crystal violet 0.4% for 15 min. The cover glass was then rinsed with more distilled water and observed under a microscope with 40x magnification.

For Scanning Electron Microscopy (SEM) observation, the cover glass was fixated with 2% glutaraldehyde and incubated at its optimum temperature for 24 h. Next, the cover glass was dehydrated with alcohol 30%, 50%, 70%, 96% and 100% for 15 min each [22]. The cover glass was then dried at 37 °C for 10 min. The specimen was then coated with gold (Au), SEM-EDS was used to examine the biofilm surface with 1000x and 2000x magnification [23, 24].

#### Gas chromatography and mass spectrometry (GC-MS) analysis

The INA metabolite was identified through GC-MS analysis using GCTrace1310 and MS ISQ LT. The sample was injected and ran through column type TG5MS for 36 min. Helium gas (99.999%) was used as a carrier gas with a flow rate of 24 mL/min and a temperature of 325 °C [25].

#### Molecular identification of INA bacteria isolates

INA Bacteria isolates were identified using 16 S rRNA gene sequencing. DNA genome was extracted using Promega Wizard Kit DNA Extraction. Specific primers, 63 F and 1387R, were used [26]. The final volume of PCR master mix consists of 1 µL of DNA template, 1 µL of each primer, 12.5 µL of GoTaq, and 9.5 µL of nuclease-free water. PCR condition was set as per following: 94 °C pre denaturation for 5 min, followed by 30 cycles of denaturation at 95 °C for 30 s, annealing at 55 °C for 30 s, elongation at 72 °C for 1 min and post elongation at 72 °C for 5 min [27]. The product was separated using 1.5% agarose gel electrophoresis at 80 V for 45 min and visualized under GelDoc. The results were then sent to Genetika Science for sequencing and then submitted to GenBank.

#### Statistical analysis

The data will be statistically analyzed using IBM SPSS Statistics 24. The analysis used was non-parametric Kruskal-Wallis on the data. The observed difference is significant if the probability obtained is less than 0.05.

## Results

### Detection of antimicrobial activity and anti-quorum sensing activity

From antimicrobial activity assay all of the INA metabolite have no antimicrobial activity toward fish pathogenic bacteria. as no clearing zone were found around the wells (Supplementary Table 1). While for anti quorum sensing activity, we found that four metabolite of INA showed anti quorum sensing activity as indicated by non-purple opaque zone around the wells (Supplementary Table 2).

### Validation of quorum sensing inhibition

The four INA isolates tested positive for Anti-Quorum sensing activity were then validated as indicated by a lower absorbance number after being treated and isolate A19 performed the best inhibition with absorbance number of 0.0335 which was much lower than 0.0721 as the positive control (Supplementary Fig. 1).

### Biofilm inhibition and destruction assay

**Fig. 1:**
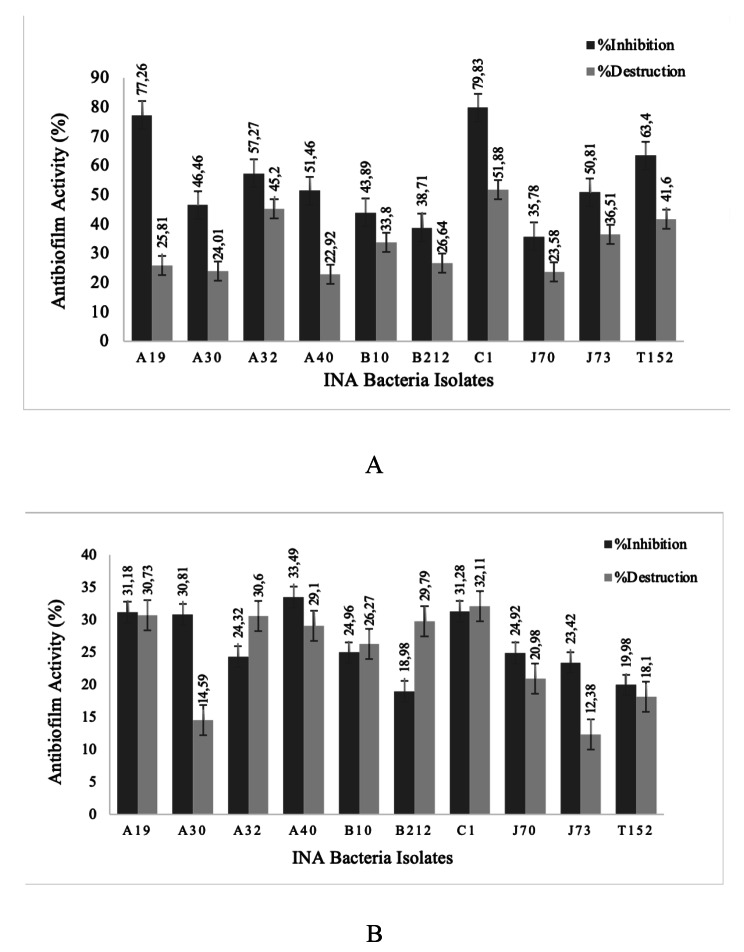
(**A**) Antibiofilm activity against *S. agalactiae* (**B**) Antibiofilm activity against *A. hydrophila*

An anti-biofilm assay was carried out for both fish pathogenic bacteria to determine the ability of INA to metabolize as an anti-biofilm agent by using 100µL of the metabolite to 100µL to the suspension of pathogenic bacteria. Figure 1 shows all bacterial metabolites that inhibit and disrupt Biofilm. Based on this assay, it is known that isolate C1 had performed the highest inhibition and destruction percentage against *S. Agalactiae* with a percentage of 79.83% and 51.88% respectively. As for isolate A40 and C1 had shown the best inhibition of 33.49% and destruction of 32.11% respectively, against *A. hydrophila.*

### Microscopic observation of biofilm

**Fig. 2:**
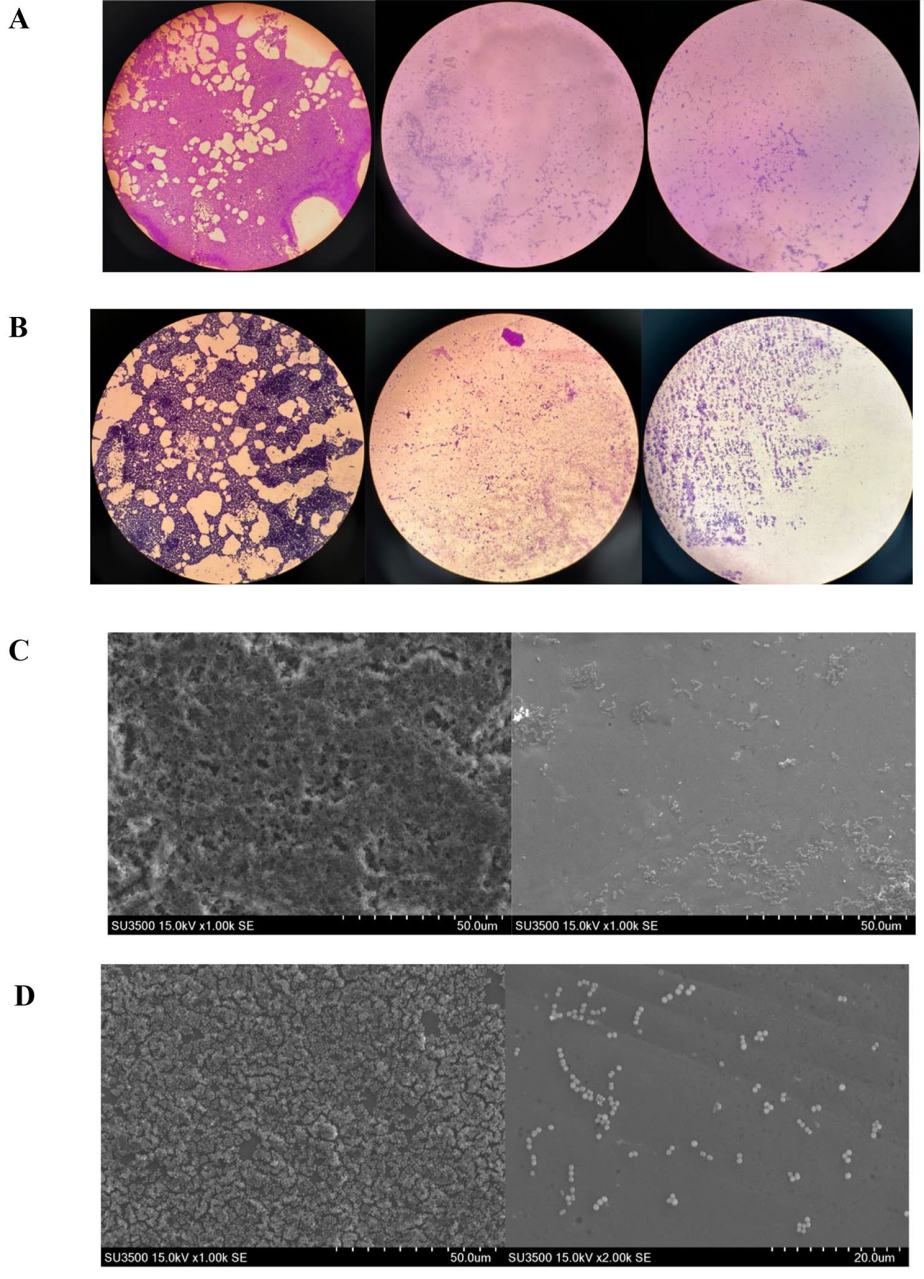
(**A**) Light microscopic observation on biofilm destruction activity of *A. hydrophila* (**Left**) control (**Centre**) A32 supernatant treatmet (**Right**) B212 supernant treatment. (**B**) Light microscopic observation on biofilm destruction activity of *S. agalactiae* (**Left**) control (**Centre**) B10 supernatant treatmet (**Right**) J73 supernant treatment. (**C**) Destruction activity on Biofilm of *A. hydrophila* through SEM observation. (**Left**) control and (**Right**) treated by supernatant of B212. (**D**) Destruction activity on biofilm of *S. agalactiae* through SEM observation. (**Left**) control and (**Right**) treated by supernatant of A32 metabolite

Microscopic observation was carried out based on antibiofilm activity. The structure of *A. hydrophila* biofilm and *S. agalactiae* biofilm formed before and after the treatment was observed using light microscopy (Fig. 2A-B) and the results were further confirmed by using SEM-EDS observation (Fig. 2C-D). The elements of each pathogenic biofilm were characterized using SEM- EDS (Supplementary Table 3).

Based on light microscopy, it was found that there was a decrease in biofilm biomass after treated with INA metabolite. As seen in Fig. 2, A. *hydrophila* biofilm when treated with isolate A32 (Fig. 2A Center) and B212 (Fig. 2A Right), had a significant decrease when compared to the positive control (Fig. 2A Left). For *S. agalactiae* biofilm, after being treated with isolate B10 (Fig. 2B Center) and J73 (Fig. 2B Right) had a biomass decrease when compared with the control (Fig. 2B Left). Further confirmation through SEM observation, as seen from the destruction activity, metabolite B212 has the ability to destroy the biofilms of *A. hydrophila*, indicated by the damaged structure of Biofilm compared to the control (Fig. 2C). Meanwhile for the observation of destruction activity, the metabolite of A32 has shown its activity to destroy Biofilm formed by *S. agalactiae* and has shown a decrease in cell biomass formed when compared to the control (Fig. 2D).

From Supplementary Table 3, untreated Biofilm of *A. hydrophila* and treated Biofilm showed differences of elements present shown by presence of Mg and Al which were not present in the untreated Biofilm. Untreated Biofilm of *S. agalactiae* showed the presence of C, O, N, Na, Si, P, K, Ca, Mg, and Al, but the treated Biofilm had lost the presence of P and N elements.

### GC-MS analysis

GCMS analysis was carried out for the selected INA metabolites, A32, B212, C1 and T152, based on their antibiofilm activity, and the results are shown in Supplementary Figs. 2–5. Each metabolite has similar components to each other, known as amino acid, fatty acid and methyl ester, as shown in (Table 1). N-hexadecanoic acid is found the highest in isolate A32 with a percentage area of 5.13%, meanwhile 9-Octadecenoic acid(z)-2-hydroxy-3-[1- (oxohexadecyl)oxyl propyl ester is found the highest in isolate B212, in isolate C1 compound 9-Octodecenoic acid(z)-2-hydroxy-1-(hydroxymethyl)ethyl ester was found the highest with percentage area of 15.70% and in isolate T152, the highest compound found was 9,12-Octadecadienoic acid with percentage area of 15.39%.

**Table 1:** Bioactive compounds of bacterial metabolite

Isolates	Compound Name	%Area
A32	Sarcosine	1.17
	N-hexadecanoic acid	5.13
	Trans-13-octadecenoic acid	2.70
B212	N-hexadecanoic acid	4.45
	9-Octadecenoic acid(z)-2-hydroxy-3-[1- (oxohexadecyl)oxyl propyl ester	15.33
	Sarcosine	1.08
C1	9-Octodecenoic acid(z)-2-hydroxy-1-(hydroxymethyl)ethyl ester	15.70
	trans-13-Octadecenoic acid	8.11
	N-hexadecanoic acid	4.19
T152	9,12-Octadecadienoic acid	15.39
	N-hexadecanoic acid	8.84
	Acetic acid	1.23

### Molecular identification of INA bacteria isolates

Eight INA bacteria isolates were identified through DNA sequencing of the 16 S rRNA gene. The results were then submitted to GenBank. It was found that all of the eight isolates identified showed similarities above 85%, isolates A19, A30, and A32 are similar to *Pantoea stewartia* with percentage similarities of 98.48%, 99.13%, and 98.56% respectively, meanwhile isolates A40, B212, and J73 are similar to *Acinetobacter* with a percentage similarities of 99.44%, 99.60%, and 99.20% respectively, and isolates B10 and J70 are similar to *Enterobacter* with percentage similarities of 98.97% and 99.05% respectively (Supplementary Table 4).

### Statistical analysis

Through SPSS statistical analysis, it is found that both inhibition and destruction assay against the two pathogens obtained 0.00 which was less than *p* = 0.05 which means that this assay was significant (Supplementary Figs. 6–7).

## Discussion

Finding of antibiofilm is very important to control aquaculture pathogenic bacteria that usually form biofilm as their survival strategy. In our study antibiofilm are explored from supernatant of ice nucleation active bacteria. As preliminary step it is important to confirm that the bioactive compound of INA bacteria have no antimicrobial activity against the tested pathogens. From Table 1 it was confirm that all of the INA strains have no antimicrobial activity. It is important to prevent false positive result of antibiofilm assays [17].

Four out of ten INA metabolites, A19, A32, A40 and B212 have shown positive results in anti-QS activity against *C. violaceum* wild type, which are indicated by the formation of a non-purple opaque zone formed around the wells. *C. violaceum* produce violacein pigment using quorum sensing mechanism is used as indicator bacteria to assessed antiquorum sensing activity [28]. *C. violaceum* wild type acts as an indicator bacterium as it can secretes a purple pigment known as violacein which is regulated through quorum sensing activity. Operon *vio* which is responsible for the production of violacein plays a crucial part, and is regulated by the presence of AHL by protein *CviI*. As for the other six isolates, it does not show QQ activity [29].

The previous test of QQ activity were conducted as qualitative assays further test were required to confirm the activity quantitatively. All positive bacterial metabolites were further tested to validate QQ activity against *C. violaceum* 026 mutant, which was indicated by the decrease in absorbance value compared to the control *C violaceum* 026. *C. violaceum* 026 differs from the wild type as it does not have the ability to produce violacein pigment due to the insertion of double transposon in Tn5. However, *C. violaceum* mutant type still has the ability to detect the presence of AHL and produce violacein [28]. The lower absorbance compared to the control indicates that the isolate can reduce violacein production as these bacterial metabolites may act as a quorum quencher. The bioactive compounds are able to inhibit QS through several mechanisms, such as inhibiting the autoinducer transcriptional regulator, degradation of autoinducer, competitive inhibition to the binding site of signal molecule receptors, or interfering with the QS signaling transport pathway [30, 31].

Ten isolates were then further investigated through biofilm inhibition and destruction assay (Fig. 1A-B), the results showed that all of the metabolites were able to inhibit or destroy biofilms. Antibiofilm activity against *A. hydrophila* biofilm performed lower than *S. agalactiae* biofilm. Their activities varied greatly between pathogens as each of the biofilms have different compositions of the biofilm matrix [32]. As seen from the results, isolates A40 and C1 showed the highest inhibition activity against *A. hydrophila* and *S. agalactiae* which were 33.49% and 79.83% respectively. Whereas isolate C1 had performed the highest in biofilm destruction assay for both *A. hydrophila* and *S. agalactiae* with the percentage of 32.11% and 51.88% respectively. The inhibition and destruction activity can differ between each other as it might be caused by the difference in EPS components and in the metabolite compound of the two types of pathogens. Moreover, the genetic regulation can vary between species and strains of bacteria [33, 34]. The INA metabolites performed lower in antibiofilm activity against *A. hydrophila* as it has a rigid cell membrane that prevents entry of any compound into the cytoplasm and also lipopolysaccharide which limits the penetration of hydrophobic compounds into the cell [35].

In a previous study, INA bacteria, *P. agglomerans*, had shown antibiofilm activity toward marine pathogen, *Vibrio* spp., this specific INA bacteria produces alpha-amylase enzyme which is able to destroy mature biofilms formed. Earlier studies conducted stated that *K. pneumoniae* has the ability to produce monosaccharides which is able to inhibit the interaction between bacteria and the surface [11, 12].

Determination using light microscopy and scanning electron microscopy showed and confirm that bacterial metabolite from B212 are able to disrupt biofilm form by *A. hydrophila*, and metabolite from A32 perfomed capability to disrupt biofilm of *S. agalactiae*. It is shown that biofilm of each bacteria are specific and might differ in their composition therefore the metabolite with antibiofilm activity is also specific for each biofilm [36].

. From Supplementary Table 3, untreated Biofilm of *A. hydrophila* and treated Biofilm showed differences of elements presence. Both treated Biofilm of fish pathogens showed a decrease in C element, which may be caused by the decreasing amount of biofilm biomass as carbon is one of the main components in forming a biofilm, polysaccharides. Nitrogen found in untreated *S. agalactiae* biofilm is known to be the main component of protein that makes up Biofilm. Inorganic elements Al, Na, P, and sulfides found, able to bind with polysaccharides which contributes to the formation of Biofilm and adhesion. In both treated Biofilm of fish pathogens, the phosphate element is a main component of extracellular DNA (eDNA) which decreased in number that may conclude the absence of eDNA from the treated Biofilm [32, 37, 38]. Other causes of cell biomass decrease may be caused by the weakening of biofilm attachment to the surface as EPS may be inhibited by organic compounds that interfere with biofilm maturation and resulted in poor adherence and stability after treatment. Organic compounds, in the form of enzymes may also destroy mature Biofilm through the process of hydrolyzing biofilm exopolysaccharide [20. Absorption capacity, pH, and optimum temperature of biofilm formation may influence the difference in distribution and total weight of the sample [32].

Each metabolite has its own antibiofilm component therefore the antibiofilm mechanism might be different. Aside from different types of compounds found, the concentration of each compound may differ from each metabolite, shown by the different performances of antibiofilm activity (Supplementary Figs. 1–5). As seen in Table 1, major compound of the isolates are sarcosine; hexadecenoic acid; octadecanoic acid; methyl ester and acetic acid. Selected isolates produced sarcosine, a type of amino acid that helped resist the attachment of both *Escherichia coli* and *Pseudomonas aeruginosa* [39]. Fatty acids such as palmitic acid (n-Hexadecanoic acid) and linoleic acid (9,12-Octadecadienoic acid) are also found in almost all of the supernatants and have been known to inhibit the formation of Biofilm in *Acinetobacter baumanii* without affecting its planktonic cells [40]. Moreover from *in silico* study, hexadecanoic acid analogs reported as potential CviR-mediated quorum sensing inhibitors in *Chromobacterium violaceum* [41] and Octadecenoic acid from the rhizospheric bacterium *Stenotrophomonas maltophilia* BJ01 also reported show quorum quenching and anti-biofilm activities [42], both of this also reported have antimicrobial activity [43]. Another bioactive compound found is methyl ester which has the potential to inhibit biofilm growth in *Pseudomonas aeruginosa* [44]. In general acetic acid is known for its antimicrobial activity, but it is also reported with the capability to destruct biofilm of both Gram-positive and Gram-negative as a liquid and as a dry salt [45].

Eight of the INA bacteria isolates were sequenced, 3 isolates, A19, A30, and A32, had similarities to *Pantoea stewartii*, a pathogenic bacteria mostly found on the leaf surface of a maize plant, that causes Stewart’s wilt disease [46]. Meanwhile isolates A40, B212, and J73 had similarities to *Acinetobacter* that causes opportunistic infections and mostly found in soil and natural water systems [47]. As for isolates B10 and J70 had high similarities with *Enterobacter genera*, that are easily found in environmental habitats, mostly from soil and water, endophytic and can be considered phytopathogenic against various plant species [48].

In previous studies, it was found that INA bacteria, *Pantoea stewartii*, is able to secrete WceF, an enzyme which is able to cleaves *stewartan* exopolysaccharide [49]. Other studies also found that aqueous extract of *Enterobacter* sp., inhibits Biofilm of staphylococcal [50]. It is also known that *Enterobacter* sp. strain 84.3 is effective in inhibiting Biofilm and disaggregating the mature Biofilm without killing the planktonic cells of *Staphylococcus aureus* and *Staphylococcus epidermidis* [51].

## Conclusions

Thus, this research demonstrates that *Pantoea stewartii, Acinetobacter*, and *Enterobacter* have a significant antibiofilm activity against Biofilm formed by *A. hydrophila* and *S. agalactiae* for both inhibition and destruction assay. More deepened research is still needed, incubation period for the antibiofilm assay may be optimized to receive better results. Therefore, the metabolite of INA bacteria has the potential to be applied to control the Biofilm of tested fish pathogens.

### Limitations

Fish pathogens were tested are limited to *A. hydrophila* and *S. agalactiae*; therefore, the bacterial metabolite is important to be tested to other pathogens related in aquaculture.

### Electronic supplementary material

Below is the link to the electronic supplementary material.

Supplementary Material 1

## Data Availability

The datasets used and/or analysed during the current study are available from the corresponding author upon reasonable request. The sequence of 16 S rRNA gene have been deposited in Genbank for isolate A19 with accession number OR128363; A30 with accession number OR131584; A32 with accession number OR131589; A40 with accession number OR131746; isolate B10 with accession number OR131747; isolate B212 with accession number OR133538; isolate J70 with accession number OR133539; and isolate J73 with accession number OR133540.

## Abbreviations

INA: Ice Nucleation Active
LB: Luria Broth
QS: Quorum Sensing
QQ: Quorum Quenching
SEM: Scanning Electron Microscope
EDS: Energy Disperse X-ray Spectroscopy
EPS: Extra Polymeric Substance
