# Supplementary material for: Ice nucleation active bacteria metabolites as antibiofilm agent to control Aeromonas hydrophila and Streptococcus agalactiae infections in Aquaculture
Source: BMC Res Notes. 2024 Jun 17;17:166. doi: 10.1186/s13104-024-06821-9 (PMC11184859; doi:10.1186/s13104-024-06821-9)
Supplement: Supplementary file 1 — Supplementary Material 1 [file 13104_2024_6821_MOESM1_ESM.docx]

**SUPPLEMENTARY FILE**

Supplementary table 1 Detection of antimicrobial results from fish pathogens.

| Isolates | 1. *hydrophila* | *S. agalactiae* |
| --- | --- | --- |
| A19 | - | - |
| A30 | - | - |
| A32 | - | - |
| A40 | - | - |
| B10 | - | - |
| B212 | - | - |
| C1 | - | - |
| J70 | - | - |
| J73 | - | - |
| T152 | - | - |

+: positive antimicrobe ; -: negative antimicrobe

Supplementary table 2 Anti-quorum sensing activity against C. violaceum wild type

| Isolates | QQ against CV WT |
| --- | --- |
| A19 | + |
| A30 | - |
| A32 | + |
| A40 | + |
| B10 | - |
| B212 | + |
| C1 | - |
| J70 | - |
| J73 | - |
| T152 | - |

+: positive quorum quenching; -: negative quorum quenching

Supplementary table 3 Total weight of biofilm element

| Element | 1. *hydrophila* (%) | *A.hydrophila*  *+*  B212 (%) | *S. agalactiae* (%) | *S. agalactiae*  *+*  A32 (%) |
| --- | --- | --- | --- | --- |
| C | 74.94 | 16.13 | 43.58 | 20.64 |
| O | 22.40 | 46.38 | 25.82 | 44.44 |
| Na | 0.94 | 6.44 | 3.95 | 6.49 |
| Si | 0.38 | 24.77 | 15.72 | 22.75 |
| P | 0.69 | - | 0.25 | - |
| S | 0.47 | 0.06 | - | - |
| K | 0.08 | 0.21 | 0.14 | 0.16 |
| Ca | 0.10 | 3.91 | 2.54 | 3.60 |
| Mg | - | 1.80 | 1.08 | 1.57 |
| Al | - | 0.30 | 0.20 | 0.33 |
| N | - | - | 6.70 | - |

Supplementary table 4 Identification of Ice nucleation active isolates

| Isolates | Identified Bacterial Strain | %Similarity | GenBank accession number |
| --- | --- | --- | --- |
| A19 | *Pantoea stewartii* strain HR3-48 | 98.48 | OR128363 |
| A30 | *Pantoea stewartii* strain HR3-48 | 99.13 | OR131584 |
| A32 | *Pantoea stewartii* isolate RON18713 | 98.56 | OR131589 |
| A40 | *Acinetobacter pittii* | 99.44 | OR131746 |
| B10 | *Enterobacter sp.* strain HSTU-Sh65 | 98.97 | OR131747 |
| B212 | *Acinetobacter baumanii* strain 2022CK-00772 | 99.60 | OR133538 |
| J70 | *Enterobacter sp.* strain M204 | 99.05 | OR133539 |
| J73 | *Acinetobacter junii* strain QLN201710OPB4 | 99.20 | OR133540 |


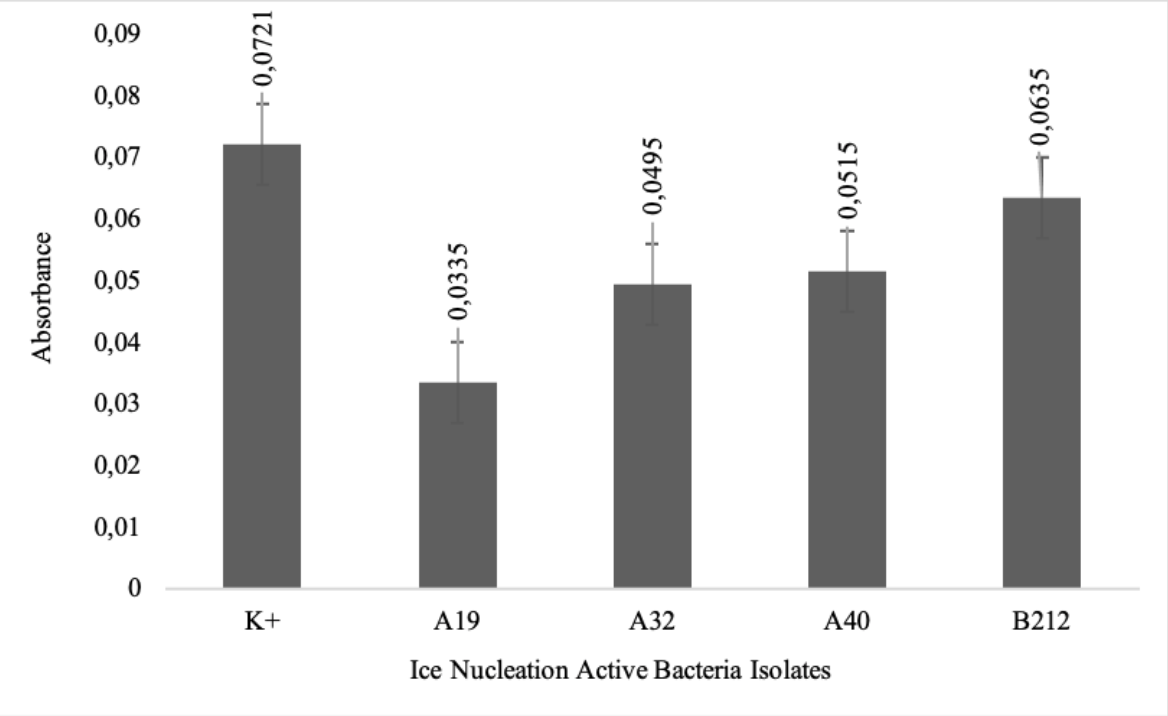


Supplementary figure 1 Validation of quorum sensing inhibition activity of 4 INA supernatant against CV 026


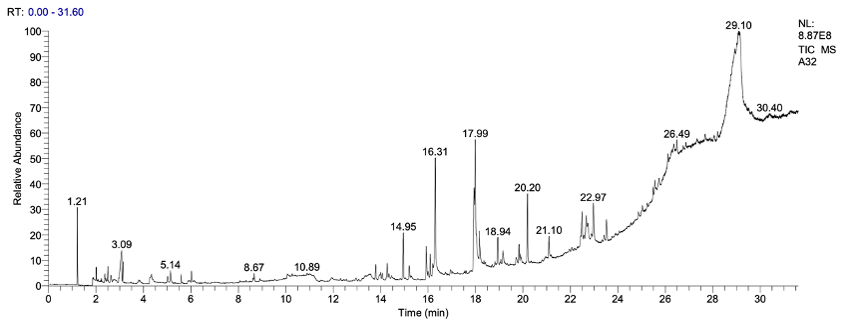


Supplementary figure 2 GC-MS graphic of isolate A32


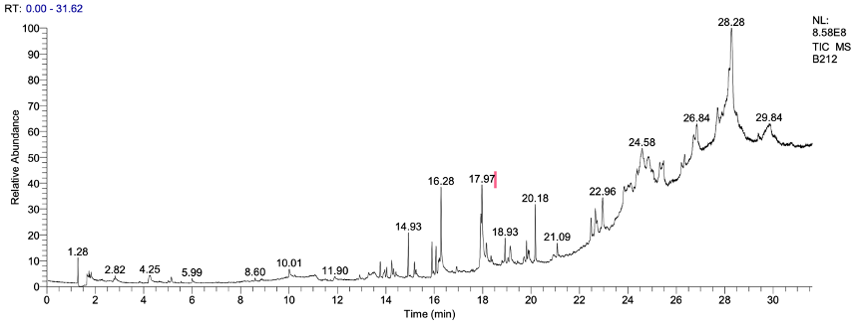


Supplementary figure 3 GC-MS graphic of isolate B212


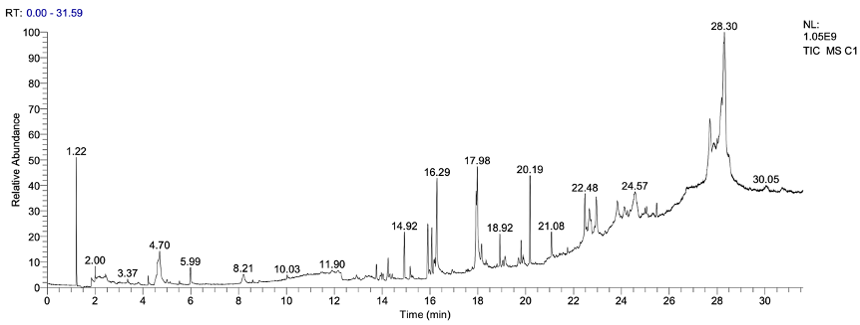


Supplementary figure 4 GC-MS graphic of isolate C1


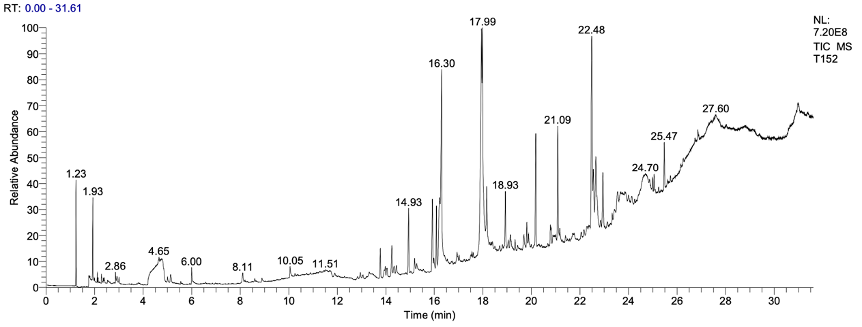


Supplementary figure 5 GC-MS graphic of isolate T152


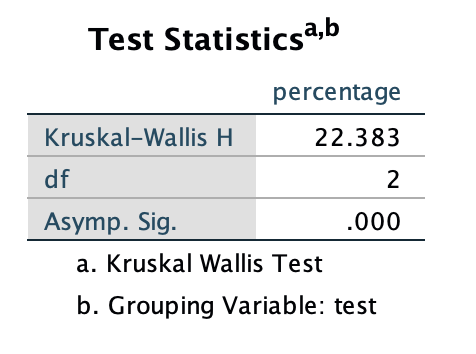


Supplementary figure 6 Statistical significance was determined with Kruskal-Wallis (**p<0.05* to *S. agalactiae* control).


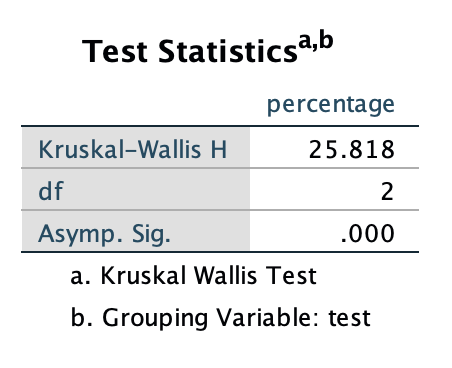


Supplementary figure 7 Statistical significance was determined with Kruskal-Wallis (**p<0.05* to *A. hydrophila* control).
